# Contamination of dental unit waterlines: assessment of three continuous water disinfection systems

**DOI:** 10.1038/bdjopen.2016.7

**Published:** 2016-12-16

**Authors:** Damien Offner, Florence Fioretti, Anne-Marie Musset

**Affiliations:** 1 French National Institute of Health and Medical Research (INSERM), Osteoarticular and Dental, Regenerative Nanomedicine, Strasbourg, France; 2 University Hospital of Strasbourg (HUS), Strasbourg, France; 3 Faculty of Dental Surgery—University of Strasbourg (UDS), Strasbourg, France

## Abstract

**Objectives:**

To assess the efficacy of three continuous water disinfection systems for dental units under real conditions of dental care.

**Design and settings:**

A prospective study carried out from 45 days to 20 months on the water microbial quality of the dental units is benefited from three different systems: two chemical treatment systems (IGN EVO/Calbenium/IGN Cartridge and Sterispray) and one physical treatment system (BacTerminator). Studied items were six dental units of the Dental Medicine and Oral Surgery Center within the University Hospital of Strasbourg (HUS), France.

**Results and disucussion:**

The IGN EVO/Calbenium/IGN Cartridge and Sterispray systems showed an immediate and long-term efficacy on contaminated dental unit waterlines. However, the first system offers ergonomic advantages (automatic system, action on the water from the water supply network). The BacTerminator system took longer to be effective and was less effective than the other two.

## Introduction

Dental medicine and oral surgery, as many medico-surgical practices, must be associated with a good infectious risk management, allowing one to control sources and vectors of cross-contamination, including water contamination.^1,2^ As water is used to cool down rotary instruments and clean the surgical site, monitoring its microbiological quality can be done using samples collected from the unit water system. These samples can reveal the presence of a planktonic or sessile bacterial flora, either in the water supply network,^2^ or as the result of a back-contamination of dental unit waterlines caused by a backflow of oral fluids when rotary instruments stop.^3–5^ A level of contamination up to 10^5^ c.f.u./ml has already been reported in literature.^6^ The elevated surface-to-volume ratio of dental unit waterlines (which are long thin pipes), as well as the presence of laminar water flow, can promote biofilm formation.^7–9^ Consequently, there is a distinct risk for the patient and the dental team, who are in direct contact with contaminated water. In Italy, the death of an 81-year-old female patient has been reported after she contracted Legionnaire’s disease due to contaminated water in the internal dental unit waterlines.^2^

In France, the microbiological quality of the output water from dental unit waterlines is not regulated. However, at the University Hospital of Strasbourg (HUS), quality standards have been set. The output water must at least meet the input water quality standards, which corresponds to the American Dental Association (ADA) requirements of 1995 (maximum heterotrophic plate count (HPC) at 22 °C of 200 u.f.c./ml),^10^ although the ADA followed the United States’ Center for Disease Control and Prevention (CDC) in 2004, recommending a maximum HPC of 500 c.f.u./ml. Furthermore, the target value of the microbiological quality of the dental units water set at the HUS meets the European Union’s standard for potable water with an HPC of <100 u.f.c./ml.^11^

If these criteria are not respected, the use of the dental chair will be suspended and remedial actions will be undertaken along with the strengthening of the maintenance protocol implementation and the use of a sheet of traceability. In some cases, remedial maintenance such as the disinfection of the internal dental unit waterlines will be undertaken as well.^12^

In 2013, 28% out of the samples collected at the Dental Medicine and Oral Surgery Center of the HUS were non-compliant. Remedial actions undertaken for these units consisted in the treatment of the internal dental unit waterlines using Dialox (a disinfectant made from peracetic acid, hydrogen peroxide and acetic acid). The efficiency level of this treatment was of 75%, which was considered as insufficient. Consequently, the Dental Medicine and Oral Surgery Center decided to carry out actions in order to increase this efficiency level. As a result, continuous water treatment systems (IGN EVO/Calbenium/IGN Cartridge, Sterispray and BacTerminator) were set on the contaminated units resisting the Dialox disinfection treatment, along with a regular inspection of sampling results.

## Materials and methods

### Scope of the continuous water treatment systems tested

The continuous water treatment systems tested were the following: IGN EVO/Calbenium/IGN Cartridge, Sterispray and BacTerminator. They have been attached upstream from the dental unit waterline. The first two systems are both chemical treatment systems, that is to say that a disinfectant is injected from an independent reservoir into the dental unit waterline. The third system is a physical treatment system, which means that the input water goes through several treatment phases, such as filtration and ionization. The water used within the dental care center is of potable quality regarding the European Union’s standard,^11^ which means that it is under microbiological monitoring to present an heterotrophic plate count (HPC) at 22 °C below 100 c.f.u./ml, an heterotrophic plate count (HPC) at 37 °C below 10 c.f.u./ml, and coliform bacteria as well as *Pseudomonas aeruginosa* below 1 c.f.u./ml.

- IGN EVO/Calbenium/IGN Cartridge (referred as IGN EVO Calbenium later in the text): This system, used in accordance with the manufacturer’s recommendations, includes an IGN device, which is an automatic dilution system diluting a liquid disinfectant concentrate called CALBENIUM in the water supply network. The IGN is attached to the water supply network going under the unit, in compliance with the manufacturer’s recommendations. A cartridge of IGN ensures the decrease of diatoms loads in water. The appropriate dose of Calbenium is automatically injected in the water flow going onto the unit shelf. The level of Calbenium is constantly monitored with an audio-visual detection system. The Calbenium solution is composed of EDTA, benzalkonium chloride, sodium tosylchloramide, allantoin, aspartame, sorbitol and spearmint flavor. Proportions of these products are not disclosed by the manufacturer.
- Sterispray has been used in accordance with the manufacturer’s recommendations and attached to the units. The system is designed as an independent water reservoir which works with compressed air and delivers water into the sprays. This is a manual filling system which must contain only either distilled or demineralized water to dilute the disinfectant product Sterispray. This product is manually added each time the system is filled. It may be necessary to fill it several times on a daily basis. The solution is composed of EDTA, benzalkonium chloride, chloramine-T, glycerin, aspartame, sorbitol, flavors and thyme essential oil. Proportions of these products are not disclosed by the manufacturer.
- BacTerminator has been used in accordance with the manufacturer’s recommendations and attached to the dental units waterlines supplied with water of hospital quality. In addition to various filtration levels and ion-exchange processes, which allow, according to the manufacturer, to ‘remove calcium from water’, an electrochemical process produces hypochlorous acid and hypochlorite. These two biocidal compounds should affect the bacteria of the input water within BacTerminator. According to the BacTerminator’s manufacturer, the output water is ‘free from micro-organisms and loaded with free residual chlorine (0.5–1 p.p.m.) that ensures biocidal action’.

### Units covered in this study

This study focused on contaminated dental units which were resistant to the internal treatment using Dialox. Consequently, samples revealing the contamination of the dental unit waterlines stand for reference samples.

Three groups of two units respectively were formed. Each group was equipped with one of the three systems studied (Table 1): the IGN EVO Calbenium system for units 1 and 2, the Sterispray system for units 3 and 4 and the BacTerminator system for units 5 and 6.

All of these dental units underwent the same daily maintenance, matching the professional guidelines^13,14^ and formalized in a specific sheet of traceability. Part of the maintenance protocol relevant to the disinfection management of the dental unit water lines is described below:

- Every morning:
  - Installation of the independent water reservoir, if required.
  - Purge of the handpiece hoses for 5 min (water sampling site).
  - Purge of the handpiece hoses for 20–30 s before unplugging and treating the handpieces.
  - Purge of the handpiece hoses once the last patient has been taken care of, for 20–30 s, before unplugging and treating the handpieces.
  - Rinse of the independent water reservoir with tap water, cleaning and disinfection using a washer disinfector at 55 °C.
- Between each patient:
  - Purge of the handpiece hoses for 20–30 s before unplugging and treating the handpieces.
  - Purge of the handpiece hoses once the last patient has been taken care of, for 20–30 s, before unplugging and treating the handpieces.
  - Rinse of the independent water reservoir with tap water, cleaning and disinfection using a washer disinfector at 55 °C.
- Every evening:
  - Purge of the handpiece hoses once the last patient has been taken care of, for 20–30 s, before unplugging and treating the handpieces.
  - Rinse of the independent water reservoir with tap water, cleaning and disinfection using a washer disinfector at 55 °C.

### Study protocol

Initial samples collection of the output water from the selected units which were resistant to the Dialox treatment.
Installation of the continuous water treatment system on these units.
Sampling the day after system installation (D+1)
Sampling 15 days after system installation (D+15)
Sampling during a period of time ranging from 40 days to 20 months after system installation.

All the samples were collected of the output water in the morning, after the purge and before the first patient. Water samples culture conditions as well as the standards used are described in Table 2.

In reference to national technical guidelines, microbiological water quality levels and interpretation thresholds of sampling results are defined^15^ and outlined in Table 3. The aim was to obtain the same water quality both for the output and input water. In this case, the sample would be considered as compliant with the requirements.

The methods used in this study did not follow those described in the EN ISO 16954 standard regarding test methods used for biofilm treatment in dental unit waterlines,^16,17^ as these methods are only applied under laboratory conditions. Yet, the aim of this study was to assess the results of the water treatment systems obtained in the actual clinical context. However, the methods used in this study met standards on several aspects:

A control group was not required, as samples collected before systems installation showed contamination of the waterlines.
Two units at least were included in each test group (Table 1).
Tests were carried out over more than 4 weeks.
Count of colonies on the culture medium was performed in compliance with the standards referenced in Table 2.

## Results

**Figure 1:**
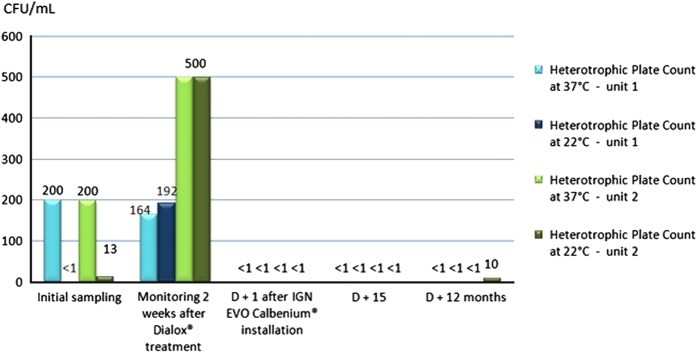
Microbiological monitoring of the output water from units 1 and 2 equipped with the IGN EVO Calbenium system.

**Figure 2:**
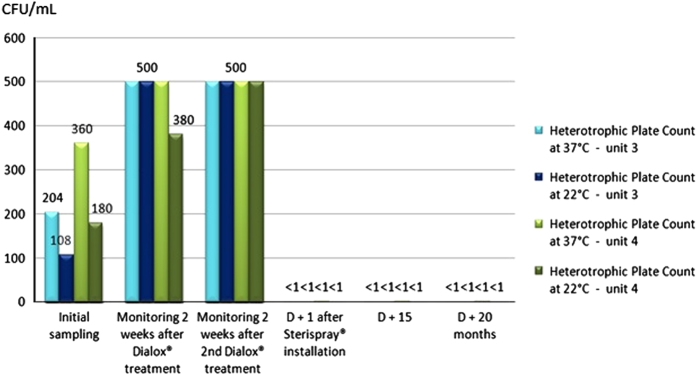
Microbiological monitoring of the output water from units 3 and 4 equipped with the Sterispray.

Concerning the units equipped with the IGN EVO Calbenium and Sterispray systems (Figures 1 and 2), primary samples collected prior to the Dialox treatment revealed levels of contamination of the output water up to 360 c.f.u./ml for the heterotrophic plate count (HPC) at 37 °C.

Samples collected 15 days after the Dialox treatment on each unit showed persistent water contamination with levels up to 500 c.f.u./ml for HPC at 37 °C and at 22 °C.

**Figure 3:**
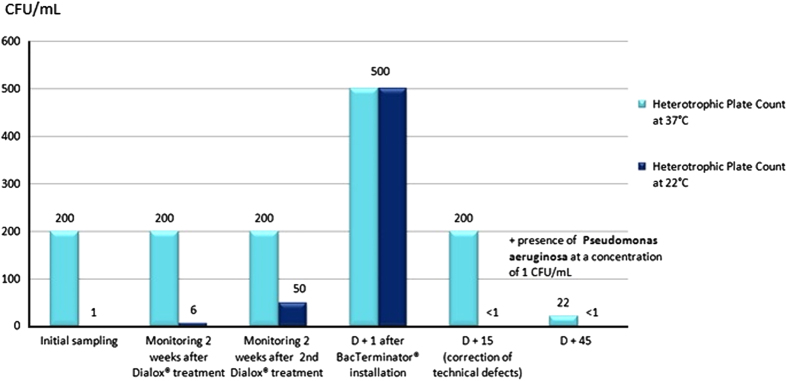
Microbiological monitoring of the output water from unit 5 equipped with the BacTerminator system, showing the correction of technical defects after samples collection at D+ 15.

**Figure 4:**
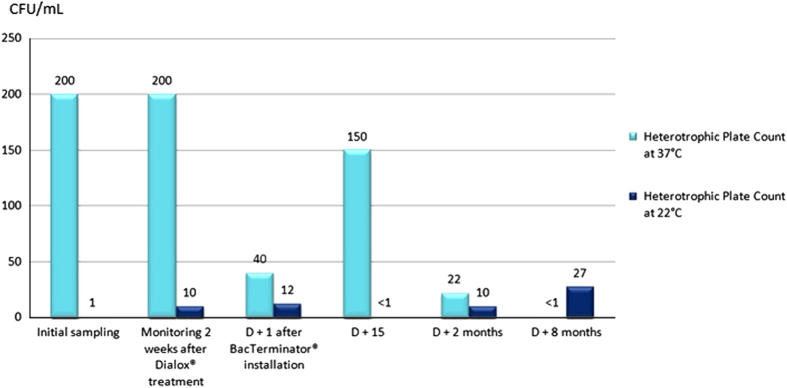
Microbiological monitoring of the output water from unit 6 equipped with the BacTerminator system.

The very next day after installation of the IGN EVO Calbenium and Sterispray systems, at D+1, samples revealed compliant results, with levels below 1 c.f.u./ml for the HPC at 37 and 22 °C. Inspection done at D+15 and D+12 months or D+20 months showed that there was no more HPC in samples, and that this compliant result was lasting (Figures 1 and 2). As for units 5 and 6 equipped with the BacTerminator system (Figures 3 and 4), primary samples collected before the Dialox treatment, and control samples collected 15 days after the treatment showed levels of contamination of the output water up to 200 c.f.u./ml for the HPC at 37 °C.

Concerning unit 5, samples collected the day after BacTerminator installation showed a non-compliant result, with a concentration of 500 c.f.u./ml of HPC at 37 °C and 22 °C. An inspection of the system revealed that it was technically malfunctioning, which consequently increased water contamination. After correction of these defects, samples collected at D+15 showed improvements, but still non-compliant results, with levels up to 200 c.f.u./ml for a HPC at 37 °C. At last, samples collected at D+45 still showed non-compliant results, as it revealed a concentration of 1 c.f.u./100 ml of *Pseudomonas aeruginosa*.

As for unit 6, the contamination level decreased at D+1 and went from 200 to 40 c.f.u./ml of HPC at 37 °C after BacTerminator installation. However, the system had no impact on the HPC at 22 °C (from 10 to 12 c.f.u./ml). At D+15, the level increased up to 150 c.f.u./ml for the HPC at 37 °C, but not for the HPC at 22 °C. It was only at D+2 months that the HPC at 37 °C decreased and went down to 22 c.f.u./ml, which, in comparison with the samples collected at D+1, represents a decrease but still is not a compliant result. Finally, at D+8 months, the sampling inspection revealed that the level of the HPC at 22 °C increased up to 27 c.f.u./ml, which remains an acceptable result, but not a compliant result.

## Discussion

The continuous water treatment systems tested in this study were installed on units from which the output water samples collected showed high contamination levels. Bacteria were resistant to routine treatments done at the Dental Medicine and Oral Surgery Center of Strasbourg.^12^ Consequently, these poor conditions constituted a suitable environment to assess the efficacy of the systems tested in this study.

Sampling results after systems installation showed the efficacy of the IGN EVO Calbenium and Sterispray systems on contaminated water in the units, on which they had an immediate and long-lasting impact. Regarding the BacTerminator system, the samples collected showed irregular results with a sudden rise of the contamination level. After 15 days, the samples were still non-compliant with the expected results. Consequently, water circulating in the waterlines still presents a potential infectious risk. A longer term study is necessary to demonstrate the possible efficacy of this system. Nevertheless, in the presence of other dental units water lines disinfection systems in the market that are proven to be efficient, and for the safety of patients, it would not be ethical to continue using systems that are of questionable efficacy.

The differences found could be explained by the mechanisms of action of the different systems. Chemical disinfection applies for planktonic bacteria and outer layers of biofilm that the water flow may remove. However, chemical disinfection does not directly impact deep layers of biofilm. Yet, biofilm bacteria are much more resistant to disinfectants than planktonic bacteria, and the biofilm could remain as a potential reservoir for bacterial contamination within the unit pipes.

Physical action of the BacTerminator system takes effect upstream the input water in the unit. Consequently, even if the water in the waterlines is supposed to be bacteria-free, it could detach residual biofilm fragments without having a disinfecting effect. Indeed, the concentration of residual biocide compounds (free chlorine) may be too low. This could explain the less satisfactory results obtained with this system, which has already been hypothesized in literature.^18^ In order to support the results, a complete removal of the biofilm from the waterlines should be considered^19^ before using continuous treatment systems. To perform this tricky operation on biofilm,^20^ the efficacy of biofilm removal solutions should be assessed, such as enzymatic detergents currently developed, to be used in association with disinfectants once biofilm has been deteriorated.

The results of our study show that it is possible to obtain a microbiological quality of the output water which is compliant with the target levels recommended, by using a water treatment system connected to the water supply network. This is the reason why we chose, at the Dental Medicine and Oral Surgery Center of Strasbourg, not to use treated water in the future (distilled, osmosis or sterile water), contrary to other health care centers.^18^ Choosing not to use treated water has the following advantages: (1) no constraints regarding the storage of bottles or cans, (2) no risk linked to the manipulation of bottles or cans, and (3) besides the results showing an equal efficacy of the IGN EVO Calbenium and Sterispray systems, the fact that the IGN EVO Calbenium system is more ergonomic must be underlined, as it offers an automatic dosing system and use only tap water (Table 1). This system presents the following advantages: no manipulation or dilution performed by the dental team, and the concentration of the product delivered in the waterlines remains unchanged.

When compared with other studies about the efficacy of dental units water lines disinfection systems, it appears that IGN EVO Calbenium and Sterispray are a part of the best systems. Indeed, a 2010 study led by RAC Chate^21^ about the efficacy of Alpron system showed compliant results (HPC <100 c.f.u./ml) for 80.9% of 52 dental units tested after a one-month utilization of the system. A 2002 study by Smith^22^ showed that compliant results (HPC <100 c.f.u./ml) were maintained on six units during 6 weeks using Alpron, but these low microbial counts were maintained for 13 weeks in only four of the six units. Finally, a 2006 study across the European Union led by Schel,^20^ in which a compliant result was HPC <200 u.f.c./ml, showed various results for different disinfection systems applied during 8 weeks. Compliant results were found in 87% of 37 dental units treated with Alpron; 74% of 26 dental units treated with BioBlue; 91% of 11 dental units treated with Dentosept; 91% of 15 dental units treated with Oxygenal; 83% of 30 dental units treated with Sanosil; 78% of 10 dental units treated with Sterilex Ultra. The results of our study show compliant results (HPC <100 c.f.u./ml) for all the dental units tested for IGN EVO Calbenium and Sterispray systems up to 12 and 20 months.

## Conclusion

The continuous water treatment systems with chemical action displayed in this study (IGN EVO Calbenium and Sterispray) showed an immediate and long-term efficacy in the treatment of contaminated dental unit waterlines. This efficacy does not seem to rely on a former waterlines treatment, and as for the IGN EVO Calbenium system, seems compatible with the use of the water from the water supply network. It therefore has organizational and financial benefits. As for the continuous water treatment system with physical action used in this study (BacTerminator), it appeared as less effective and took longer to be effective. These differences could be explained by the presence of biofilm in the waterlines before installation of the systems. A full treatment of biofilm before systems installation should be considered, according to the mode of action of the continuous water treatment systems.

## Figures and Tables

**Table 1:** Water treatment systems assigned to the units groups

*Units*	*1 and 2*	*3 and 4*	*5 and 6*
System	IGN EVO Calbenium	Sterispray	BacTerminator
Type of water	Water of hospital quality	Distilled water	Water of hospital quality
Dosing system	Automatic	Manual	No dosage

**Table 2:** Culture conditions of the output water samples collected from the unit and standards used^11^

*Microorganisms sought*	*Volume analyzed*	*Maximum storage duration before analysis (h)*	*Storage temperature (°C) before analysis*	*Samples seeding conditions*	*Standards*
HPC at 22 °C	1 ml	12	5±3	72 h at 22 °C on agar PCA by inclusion or filtration	ISO 6222^23^
					
HPC at 36 °C	1 ml	12	5±3	48 h at 36 °C on agar PCA by inclusion or filtration	ISO 6222^23^
					
Coliform bacteria and *E. coli*	100 ml	6	Ambient (⩽25 °C)	24 h at 36 °C on agar TTC by membrane filtration	ISO 9308-1^24^
	100 ml	24	5±3	2nd inspection after 48 h	
					
Enterococci	100 ml	6	Ambient (⩽25 °C)	48 h at 36 °C on Slanetz agar by membrane filtration	ISO 7899-2^25^
	100 ml	24	5±3		
					
*Pseudomonas aeruginosa*	100 ml	Undisclosed	Undisclosed	48 h at 36 °C on cetrimide agar by membrane filtration	ISO 16266^26^

Abbreviations: HPC, heterotrophic plate count; PCA, plate count agar=agar used to count revivifiable aerobic mircroorganisms; TTC, tergitol=medium used for the search and count of coliform bacteria.

**Table 3:** Levels of microbiological water quality and interpretation of the output water sampling results from the unit^11^

*Results*	*Interpretation*
• HPC at 22 °C ⩽100 c.f.u./ml • HPC at 37 °C ⩽10 c.f.u./ml • Absence of pathogenic germs	• Results COMPLIANT with the expected values
	
• HPC at 22 °C >100 u.f.c./ml and <200 u.f.c./ml • HPC at 27 °C >10 u.f.c./ml and <30 u.f.c./ml • Absence of pathogenic germs	• ACCEPTABLE results The unit can be used. A new sampling procedure is scheduled at D+3
	
• HPC at 22 °C **⩾**200 u.f.c./ml • HPC at 37 °C ⩾30 u.f.c./ml or • Presence of pathogenic germs: ✓ *Pseudomonas aeruginosa* ✓ Enterococci ✓ *Escherichia coli*	• NON COMPLIANT results SUSPEND THE USE of the unit and initiate Dialox treatment. A new sampling is scheduled at D+15 after Dialox treatment, until results are compliant

Abbreviation: HPC, heterotrophic plate count.
